# Gender Differences in Suicidal Behavior in Korea

**DOI:** 10.4306/pi.2008.5.1.28

**Published:** 2008-03-31

**Authors:** Ji-Won Hur, Bun-Hee Lee, Sung-Woo Lee, Se-Hoon Shim, Sang-Woo Han, Yong-Ku Kim

**Affiliations:** 1 Department of Psychiatry, Korea University College of Medicine, Ansan, Korea.; 2 Department of Emergency Medicine, Korea University College of Medicine, Ansan, Korea.; 3 Department of Psychiatry, Soonchunhyang University Cheonan Hospital, Cheonan, Korea.; 4 Department of Psychiatry, Soonchunhyang University Seoul Hospital, Seoul, Korea.

**Keywords:** Suicide, Gender, Depression, Risk factor, Korea

## Abstract

**Objective:**

To examine gender differences in the characteristics of suicidal behavior in South Korea.

**Methods:**

Between August 2003 and December 2006, 344 suicide attempters (116 men, 228 women) participated in this study. The attempters were interviewed using the Structured Clinical Interview for DSM-IV Axis I Disorders (SCID-I), and the lethality of the attempt was measured using the Lethality Suicide Attempt Rating Scale-II (LSARS-II) and Risk-Rescue Rating Scale (RRRS).

**Results:**

Significantly more women than men were admitted to emergency rooms due to attempted suicide during the study period. The male attempters were older and had a higher rate of employment than the females. Depression was the most common psychiatric disorder in both genders. The lesion/toxicity scores of the RRRS indicated that the male suicide attempters used higher doses or more toxic agents than the female attempters. The most common methods of suicide were ingestion and cutting in both sexes. Although there were significant gender differences in the RRRS risk score and RRRS total scores, there was no gender difference in the LSARS-II scores, which suggests that patients of both sexes share a similar ambivalence regarding suicide completion or death.

**Conclusion:**

Our study should be understood within the context of the specific cultural background of South Korea. We found that males and females use similar methods when attempting suicide and share a similar ambivalence regarding the outcome of the attempt; however, there was a difference in severity of the attempt between the two groups. Our findings may aid in the identification of more effective methods of intervention to prevent suicide.

## Introduction

Assessments of the degree of suicide lethality and investigations into relevant risk factors have important practical implications for the prevention and treatment of suicidal behavior. To study and treat suicidal and other self-injurious behaviors, researchers must examine each of the many factors involved in the behavior.^1^ Epidemiological studies of individuals who attempted suicide have suggested that clinical risk factors have important implications for the severity of the act.^2^,^3^ In other words, some people at risk of suicide are in greater need of preventive care than others as there are clear differences among individual attempters.^4^ Investigations of these differences can allow us to determine the best possible treatment and prevention strategies.^5^ Although previous studies have reported that the factors associated with suicide lethality are multifaceted,^3^ gender is a well-established and well-understood risk factor for lethality.

Because gender has been determined to be a socio-demographic predictor of suicidal behavior,^6^-^8^ gender difference should be taken into consideration in the prevention and management of suicide attempts. A number of studies from many different countries have identified gender differences in the rates and the seriousness of attempted suic ide. While the attempted suicide rates are higher in women, suicide completions are more frequent among men. These findings suggest that men tend to use more risky and more aggressive methods when attempting suicide than women.^9^ While there is converging evidence that gender is a clinically important factor for suicide lethality, the question of which specific variations are due to gender still remains.

Many researchers have investigated on the effects of suicide intent on a variety of factors. The majority of studies have suggested that male attempters have a stronger desire to commit suicide and use more lethal methods, and these severe intentions and methods cause men to engage in more fatal behaviors and complete suicide at a higher rates.^5^,^6^ However, the approaches used in many of the previous studies have several limitations. First, although there has been a point of convergence about the meaning of the words 'intent' and 'severity', the two terms have been used together in several studies. According to Beck, Schuyler and Herman,^10^ a suicide attempt can be defined by two separate criteria: (a) the nature and severity of the self-injury and (b) the degree of intent to die at the time of the act. Several studies have pointed out that suicide intent does not necessarily reflect the severity of the suicidal behavior, or there is little correlation between the intent and the seriousness of the suicide attempt.^11^-^13^ That is, even if two individuals have a similar level of intent to die, the risk of death in each individual may differ because the environment and suicidal methods are incredibly diverse for each attempt.^14^ Considering that the relationship between the risk of attempted suicide and the attempter's intent are not apparent, a study focusing on the "nature and severity" rather than the "degree of intent" may lead to a better understanding of the causes of gender difference in lethality. The second limitation of the previous studies was that they did not consider cultural diversity. Many studies conducted in Western countries found that women tended to use poison when attempting suicide, while men tend to use firearms.^15^,^16^ However the methods of suicide in Northeast Asia, including South Korea, may differ from those in the West because it is illegal for private citizens to possess firearms in these countries.

To our knowledge, few studies have investigated the gender differences in the circumstances surrounding attempted suicides, suicidal behavior, or the medical severity of the attempts. Moreover, the majority of studies on suicidality have been conducted in Western countries, even though over two-fifths of the world's suicides are committed in Asia.^17^ South Korea has the highest recorded suicide rate of the Organization for Economic Cooperation and Development (OECD) member countries.^18^ When comparing the changes in suicide rates, the rate of suicide in South Korea has risen by an average of 1% annually over the past 10 years, well surpassing Japan's 0.44% annual rate of increase.

### Aims of the study

As suicide is a multicausal phenomenon, gender difference should be taken into account when planning intervention strategies and therapy for individuals who attempt suicide. We aimed to explore the gender differences in the behavioral and circumstantial factors that may affect the medical seriousness of the suicide attempts and to compare the characteristics of the suicidal behavior itself, such as the agent used, lesions, accessibility to rescue and delay until discovery, and not just will or intent, between the male and female groups.

## Methods

### Sample

The subjects consisted of 344 patients (116 males and 228 females) between the ages of 15 and 82. Participants were recruited after being admitted to one of three emergency rooms following an attempted suicide between August 2003 and December 2006. The hospitals participating in the study were Korea University Ansan Hospital, Soonchunhyang University Seoul Hospital and Cheonan Hospital. The exclusion criteria were 'refusal to participate' and 'the existence of any medical risk posed by psychiatric interview'. During the study period, 375 patients were referred for participation in this study. Thirty-one of these patients refused to be interviewed and five were excluded due to significant medical impairments that prevented them from being interviewed. The 31 patients that declined participation gave the following reasons: they did not feel like participating, they did not feel any need to be interviewed, or they felt unwell due to vomiting, nausea, bleeding, etc. Each of the 344 patients gave informed consent to be interviewed, and psychiatric interviews were conducted on each patient between 1 and 24 h after admission to the emergency room, once their medical condition was stabilized and any immediately required treatment had been completed. The interviews and scales were administered in the emergency room or in the intensive care unit by extensively trained psychiatrists and clinical psychologists who were monitored and supervised on a weekly basis by the corresponding author of this study. All background data from the patients were collected from the interview data as well as the patient charts. All patients underwent a semi-structured interview for the purpose of investigating their socio-demographic characteristics. The demographic data on these patients are summarized in Table 1. Widely-used instruments that rate the leth ality of the behaviors were chosen for use in the present study. These instruments were used to assess the multiple variables involved in the attempts, including the lethality and risk factors. A clinical diagnosis was made using Diagnostic and Statistical Manual of Mental Disorders IV (DSM-IV) criteria. This study was approved by the ethics committee at each medical center.

### Instruments

#### Structured Clinical Interview for DSM-IV Axis I Disorders

Each patient's history of psychiatric illness was assessed using the Structured Clinical Interview for DSM-IV Axis I Disorders (SCID-I).^19^ In addition, information on the patient's family history, including suicide attempts, was investigated using structured questionnaires. Each interview lasted for about 45 min or less.

#### Lethality Suicide Attempt Rating Scale-II

The Lethality Suicide Attempt Rating Scale (LSARS)^20^ is an 11-item scale (0="death is an impossible result" to 10="death is almost certain"). Each point on the scale has comprehensive descriptive anchors that incorporate both the lethality of the means and the context or circumstances of the event. This scale also offers an appendix listing drugs and chemicals with lethal ranges of ingestion (in caps, tabs, ounces) according to body weight. Almost two decades have passed since the first edition of the LSARS was published, and the updated version (LSARS-II) contains a completely revised and redesigned table of drugs and chemicals based on the more current medications and more recent data on the minimum lethal doses in humans.^21^ Because the range of lethality is rather wide, we classified lethality as mild, moderate, or severe according to the corresponding items in Smith's article: (a) physical condition following episode, (b) lethality of the method used, and (c) the highest level of medical treatment needed.

#### Risk-Rescue Rating Scale

The Risk-Rescue Rating Scale (RRRS) was administered to assess the lethality of the suicide attempt.^22^ The Weisman and Worden's RRRS system is a descriptive and quantitative method for assessing the lethality of suicidal acts. According to this system, lethality can be expressed as a ratio of 5 risk and 5 rescue factors that are operationally defined, weighted, and then scored. The RRRS has 5 questions for risk assessment, and they reflect the medical danger of the attempt by assessing the agent used, impaired consciousness, lesion/toxicity, reversibility, and treatment required. The 5 rescue questions of the RRRS pertain to the location of the event, person initiating the rescue, probability of discovery by a rescuer, accessibility to rescue, and delay until discovery. In this system, each of the 5 risk factors is rated on a scale of 1 to 3, and the total is then converted to an overall risk score ranging from 1 to 5. Similarly, each of the 5 rescue factors is rated on a scale of 1 to 3, and the total rescue points are converted into a rescue score ranging from 1 to 5. High risk ratings reflect serious injury, and high rescue ratings reflect a high probability of intervention. RRRS score is determined using the formula [risk score/(risk score+risk score)]×100. The RRRS total score ranges from a low of 17 (representing a low risk score of 1 and a high rescue score of 5) to a high of 83 (representing a high risk score of 5 and a low rescue score of 1). If an attempter's risk score was 1, and their rescue score was 5, the RRRS score would be 17 [{1/(1+5)}×100=17]. This measurement provides the data on the various aspects of the attempt and on the strength of planning.^23^

### Statistical analysis

Socio-demographic variables, such as age, gender, education level, socio-economic status, marital status, and employment, were compared between men and women using χ^2^ tests and t-tests. Differences in LSARS scores, items of the RRRS, and psychiatric diagnosis between the 2 groups were analyzed using χ^2^ tests and a t-test. In addition, the correlations between risk score, rescue score and RRRS total score were analyzed to determine the relationship between scores above suggested. All statistical analyses were done using SPSS version 12.0. The level of significance was p<.05 for all analyses.

## Results

### Socio-demographic characteristics

The 344 subjects consisted of 116 (33.7%) men and 228 (66.3%) women. All socio-demographic variables are shown in Table 1. The mean age of the men (43.27±17.70 yrs) was significantly older than that of the women (36.68±13.36 yrs, t=3.527, df=184.356, p=.001). A significantly higher proportion of men than women were employed in jobs outside of the home, and the occupational fields were diverse (χ^2^=55.140, df=9, p<.001). There were no significant differences in education level, marital status, living alone, religion, socioeconomic status, family psychiatric history, and family history of suicide.

### Psychiatric diagnosis

There were no significant differences in the rates of any psychiatric diagnosis between men and women (Table 2) (χ^2^=12.001, df=7, p=.101). Depression was the most common mental illness in both gender groups (68.1% in men and 77.6% in women). The prevalence of a family history of mental disorder was not statistically different between the gender groups.

### Previous history of mental illness or suicide attempt

The difference of 'number of previous suicide attempts' was insignificant: 74 patients (16 men; 58 women) had a previous history of one suicide attempt, and 45 patients (13 men; 32 women) had made 2 previous attempts (Table 1). In addition, 3.5% of the women had made 3 or more attempts at suicide. There was no significant difference in the family psychiatric history or the rates of attempted suicide among family members.

### Risk-Rescue Rating Scale and Lethality Suicide Attempt Rating Scale-II

There were no significant gender differences in the agent used, impaired consciousness, reversibility, treatment required, location, person initiating the rescue, probability of discovery by a rescuer, accessibility to rescue, or delay. However, there was a significant difference in the lesion/toxicity category of the RRRS (Table 3). There were no significant gender differences in the rescue factors of the RRRS (Table 4). Gender differences were observed in the risk total RRRS scores, but not in the majority of risk factors and not in the total rescue score of the RRRS (Table 3 and 4). The risk score suggests that the suicide attempts made by men were more lethal (mean=2.39, S.D.=1.10) than those made by women (mean=2.12, S.D.=1.03, t=2.22, p=.027). The RRRS total score also shows differences in suicide risk between gender groups (men, mean=37.18±14.62; women, mean=34.00±13.60; t=1.98; p=.048).

Table 5 shows the significant positive correlations between the risk score, rescue score, and the RRRS total score. There was no significant difference in LSARS-II score between the groups (Table 6). Approximately half of the patients, both male and female, had an ambivalent intent to die (LSARS-II score=2.00-3.50).^21^

## Discussion

There were significantly more women than men in this study, and the men were older than the women. Depressive disorder was the most common mental illness in both gender groups. There were significant gender differences in the lesion/toxicity factor of the suicide attempts, even though ingestion, cutting, and stabbing were the most frequently used methods by both males and females.

There were several important findings of the present study. First, the rate of suicide attempts was higher in the female subjects than in the male subjects, which is in agreement with the findings of many previous studies.^24^-^26^ Furthermore, the female suicide attempters were younger than the males, and a significantly greater number of men had jobs outside of the home when compared with the women. These results confirm the findings of Aghanwa^27^ and Kumar,^6^ respectively. In contrast, there were no gender differences in demographic variables, such as education level, marital status, whether living with family or not, socioeconomic status, religion, medical illness, family psychiatric history, number of previous suicide attempts, and family history of attempted suicide. Second, depression was the most common mental illness among the suicide attempters, both male and female. The high prevalence of depression supports the findings of previous studies, which have shown that depressive disorder is the psychiatric diagnosis with the largest contribution to suicide attempts.^28^-^33^ Third, there was no significant gender difference in the, 'agent used' risk factor of the RRRS: both men and women chose ingestion as the most common method. On the other hand, the 'lesion/toxicity' risk factor was more severe in men because the male attempters tended to use a pesticide instead of a common drug or tried to use a higher dose. This finding agrees with previous findings that male suicide attempts are more often fatal than female attempts.^34^-^36^ Marks and Stokes^37^ wrote that availability, familiarity, and cultural acceptability play a role in the choice of suicide method. A number of previous studies from many different countries have supported their opinions.^38^-^40^ The findings of our study also support those of Marks and Stokes,^37^ and these characteristics reflect the cultural background of South Korea. The fact that the possession of firearms is restricted in South Korea explains why the results of our study differ from those of Western studies where firearms are available. Fourth, although there were no gender differences in the four RRRS risk factors (except for 'lesion/toxicity'), the rescue score, or the five rescue factors, the total risk score, there were gender differences in the 'lesion/toxicity' subscore and the RRRS total score. There were no significant gender differences in the total rescue score and five rescue factors, but there were significant gender differences in the total RRRS score. This finding demonstrates that there was a close relationship between the risk score and the RRRS total score (Table 6). These results are in agreement with those of the previous research showing that the risk score had a greater influence on the RRRS total score than the rescue score,^20^ and they support the findings of a previous study that indicated that the risk and the rescue scores are not clinically equivalent.^12^ Finally, unlike the RRRS scores, there was no gender difference in LSARS-II scores. The finding that the most common LSARS-II score was between 2.00 and 3.50 in both genders suggested that both male and female attempters had feelings of ambivalence toward suicide completion. We could consider the difference of intent as a factor in the discrepancy between the results of the RRRS and LSARS-II because the concept of lethality on the LSARS-II is based on the isolation, timing, precautions against intervention, seeking help during or after the attempt, notes and planning, which is rather different from the concept of lethality on the RRRS. Plutchik et al.^12^ suggested that the lethality measured by LSARS-II reflects the 'intention to commit suicide'. That is, the LSARS-II is related to the attempter's intent,^14^ while the RRRS score is related to the fatality of the behavior. Thus, it is not surprising that the LSARS-II, which is an indication of suicidal intent, showed no clear distinction between men and women,^13^ while a difference in suicidal severity existed nonetheless.

The limitations of the present study are as follows. First, generalizations about suicidal intent should not be made based on the findings of the LSARS-II alone because the semi-structured interview carried out in this study did not include any other questionnaires investigating the suicide intent due to the restrictions on what can be administered to patients in the emergency room. Second, the sample population consisted of on living patients, that is, survivors. This means that there might be a selection bias regarding the gender differences in severity because male attempters were more likely to have died before hospitalization as a result of making more serious attempts. Finally, the sample was not based on the general population of suicide attempters because only suicide attempters who were admitted to three hospitals in Gyeonggi-do and Seoul were included.

The most important achievement of this study is that we thoroughly investigated the environmental factors and behaviors associated with the suicide attempt itself by interviewing the suicide attempters as soon as possible after the attempt. Suicides are the result of the interaction of multiple factors.^16^ Therefore, suicide prevention services ought to be based on epidemiologic studies of suicidal behavior and mortality in order to offer the best possible therapy for individuals with some intent to die in a specific environment or cultural milieu. The effectiveness of services for the prevention of attempted suicide is imprecise. We hope that our study will be applied to the understanding of medically serious suicide attempts in a non-Western culture and that they will help to improve the clinical care of suicide patients.

## Figures and Tables

**TABLE 1:**
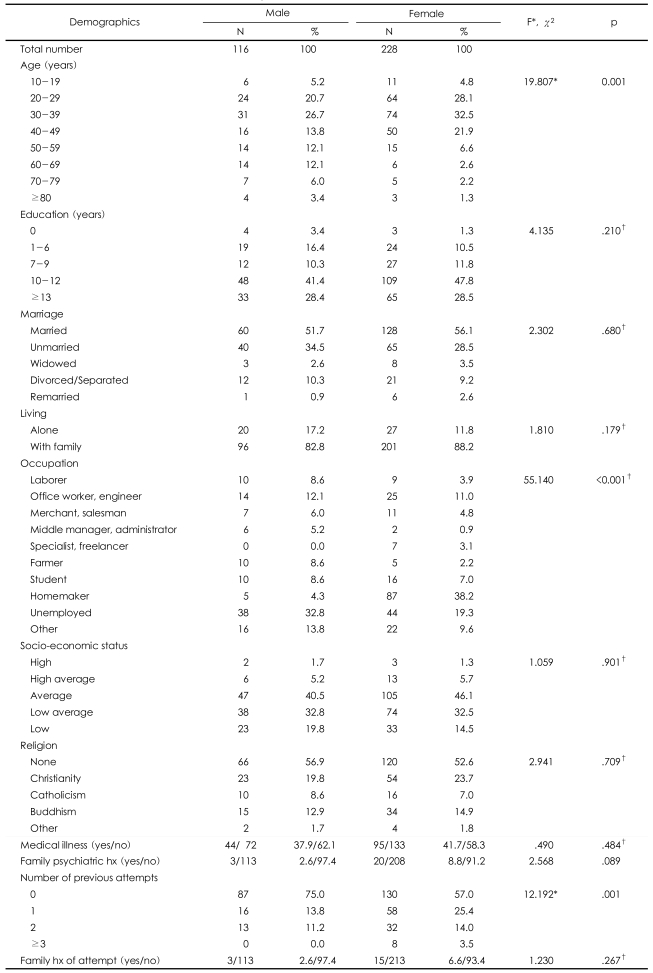
Demographic characteristics of suicide attempters

^*^levene's test, ^†^Fisher's exact probability test (p<.05).

**TABLE 2:**
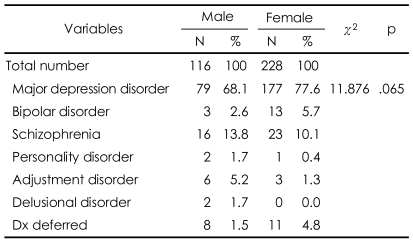
Psychiatric diagnoses of suicide attempters

Non-significant. Dx: Diagnosis

**TABLE 3:**
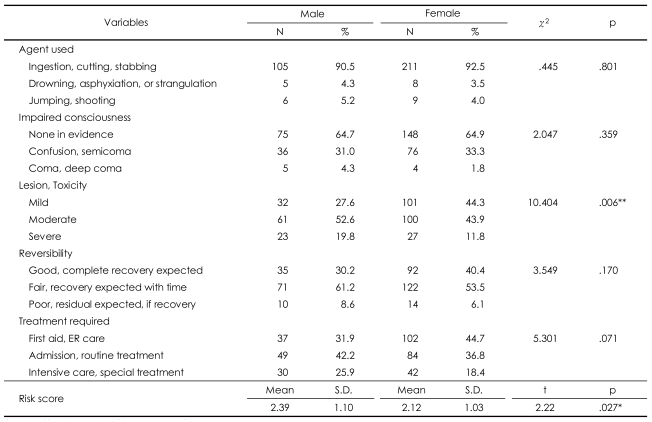
RRRS risk factors in suicide attempters

^*^p<.05, ^**^p<.01. RRRS: Risk-Rescue Rating Score

**TABLE 4:**
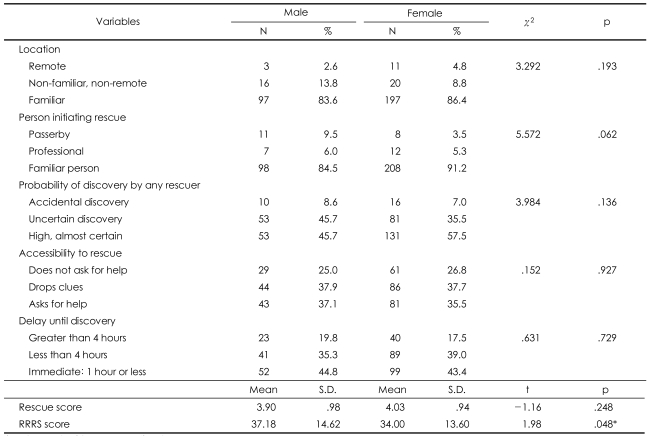
RRRS rescue factors in suicide attempts

^*^p<.05. RRRS: Risk-Rescue Rating Score

**TABLE 5:**
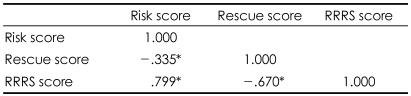
Correlation between RRRS risk score, rescue score and total RRRS score

^*^p<.01. RRRS: Risk-Rescue Rating Score

**TABLE 6:**
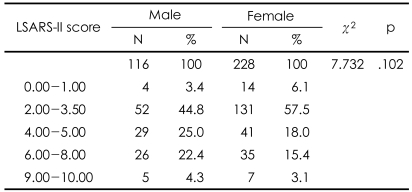
Gender differences in lethality based on LSARS-II scores

Non-significant. LSARS-II: Lethality Suicide Attempt Rating Scale-II
